# Stereology of the Thyroid Gland in Indo-Pacific Bottlenose Dolphin (*Tursiops aduncus*) in Comparison with Human (*Homo sapiens*): Quantitative and Functional Implications

**DOI:** 10.1371/journal.pone.0062060

**Published:** 2013-05-14

**Authors:** Brian Chin Wing Kot, Thomas Yue Huen Lau, Sammy Chi Him Cheng

**Affiliations:** 1 School of Nursing, The Hong Kong Polytechnic University, Hung Hom, Kowloon, Hong Kong SAR, China; 2 Department of Health Technology and Informatics, The Hong Kong Polytechnic University, Hung Hom, Kowloon, Hong Kong SAR, China; Texas A&M University-Corpus Christi, United States of America

## Abstract

The mammalian thyroid gland maintains basal metabolism in tissues for optimal function. Determining thyroid volume is important in assessing growth and involution. Volume estimation is also important in stereological studies. Direct measurements of colloid volume and nuclear-to-cytoplasmic ratio of the follicular cells may provide important information about thyroid gland function such as hormone storage and secretion, which helps understand the changes at morphological and functional levels. The present study determined the colloid volume using simple stereological principle and the nuclear-to-cytoplasmic ratio of 4 Indo-Pacific bottlenose dolphins and 2 human thyroid glands. In both dolphin and human thyroid glands, the size of the follicles tended to be quite variable. The distribution of large and small follicles within the thyroid gland was also found to be random in both the dolphin and human thyroid gland; however, the size of follicles appeared to decrease as a function of increasing age in the dolphin thyroid gland. The mean colloid volume of the dolphin thyroid gland and human thyroid gland was 1.22×10^5^ µm^3^ and 7.02×10^5^ µm^3^ respectively. The dolphin and human subjects had a significant difference in the mean colloid volume. The mean N/C ratio of the dolphin thyroid follicular epithelia and human follicular epithelia was 0.50 and 0.64 respectively. The dolphin and human subjects had a significant difference in the mean N/C ratio. This information contributes to understanding dolphin thyroid physiology and its structural adaptations to meet the physical demands of the aquatic environment, and aids with ultrasonography and corrective therapy in live subjects.

## Introduction

The thyroid gland is unique to the vertebrate endocrine system in that it stores its secretory products, thyroid hormones (THs) extracellularly. This gland is among the most highly vascularised endocrine glands in mammals and appears to be one of the oldest vertebrate endocrine glands from a phylogenic perspective [1]. The mammalian thyroid gland maintains basal metabolism in tissues for optimal function [2]. The thyroid gland synthesizes THs and stores both thyroxine (T4) and tri-iodothyronine (T3), which are important mediators of metabolism, and maintenance of homeostasis in response to environmental demands. [3]. Thyroid gland function is regulated by the hypothalamic-pituitary-thyroid (HPT) axis. Thyroid-stimulating hormone (TSH) from the pituitary gland stimulates synthesis of T4. First, the amino acid tyrosine incorporates to a glycoprotein called thyrogloblin (Tgb) which when iodinated, links to form T4. Some T4 are partially deiodinated to form the more active thyroid hormone T3 prior to release from the thyroid gland and metered into circulation to meet metabolic needs [4],[5].

Thyroid hormones influence many aspects of reproduction, growth, differentiation and metabolism. In mammals, thyroid hormones relate directly to energy intake; the responses are rapid and indicate a continuous regulation of metabolism in relation to caloric supply [6]. Thyroid hormones affect mammalian metabolism by a thermogenic action, in which carbohydrate, lipid and protein metabolism can be accelerated in tissues and thus, increase the amount of metabolic heat produced at a given time [1]. Thyroid hormones are also indispensable for normal growth and development. Altered thyroid hormone concentrations at critical developmental periods may be of special concern especially during early stage of life [3],[7],[8]. Thyroid hormones affect the metabolism of proteins, lipids, and carbohydrates.

Early investigators were impressed by the large size of cetacean thyroid glands, and special attention was then drawn mainly to the ratio of thyroid gland weight to body weight [9]–[12]. This led the assumption that cetacean metabolic rates likely exceeded those predicted by Kleiber's law [5], which presumes that the volume of the gland reflects the amount of hormone released into circulation [13]. Ridgway and Patton [12] reported that Atlantic bottlenose dolphin (*Tursiops truncatus*) had over twice the basal metabolic rate of man [12]. Irving et al [14] also showed bottlenose dolphins (*Tursiops truncatus*) have a high metabolic rate. However, later studies showed that increased metabolic rate did not correlate with thyroid gland weight [15]. Large reserves of thyroid hormones were found in beluga (*Delphinapterus leucas*) thyroid gland, with marked seasonal elevation of circulating thyroid hormones during summer, which might help mobilizing the beluga subcutaneous fat stores for somatic growth in warm estuaries[5],[10].

The functional components of the thyroid gland are the individual thyroid follicles, which consists of thin cuboidal and occasionally columnar epithelia, arranged as a single layer surrounding a lumen of colloid [1]. The variably sized and shaped follicles are separated by connective tissue in which the blood and lymph vessels and nerves are carried. In the normal state, the follicles are filled with homogeneous colloid. The epithelial cells vary in size and number dependant on the activity of the gland. In the hyperactive state, hyperplasia or abnormal increase of epithelial cells may occur [16]. Previous histological examination of the thyroid gland in marine mammal revealed no significant difference from the typical mammalian arrangement [12]. From a morphological point of view, the volumetric fraction and activity of different histological components in the mammalian thyroid gland (follicular cells, C-cells, colloid, and interstitial tissue) changed considerably in the course of development [17]. Phocids [9],[18],[19] and cetaceans [9]–[11] have shown a marked variation in the apparent levels of activity of thyroid follicular cells at various times throughout the life history.

Thyroid gland abnormalities in human patients can be clinically assessed by a number of procedures and tests. However, each diagnostic test has limitations when applied to dolphin subjects [20]. Clinical examination of the dolphin's thyroid gland is not possible due to the presence of thick blubber and prominent sternohyoideus muscles. Although analysis of serum concentrations of thyroid hormones can provide valuable insight to any thyroid function, repeated sampling may result in the loss of voluntary behaviour of the dolphin subject as well as damage skin and blood vessels [21]. Therefore this method cannot be used repeatedly to monitor thyroid gland physiology in live dolphins.

Kot et al. [22] was the first to establish a reliable and standardised ultrasonographic imaging protocol for dolphin thyroid gland assessment in a captive population of Indo-Pacific bottlenose dolphins (*Tursiops aduncus*). Individual differences in the ultrasonographic appearance and volume of the thyroid gland were noted in this species [23]. Different cetacean species have variations in thyroid gland morphology and function, which are associated with demographic variables, physiological cycles and health status [10],[11],[23]–[25]. Determining volume is important in assessing the development and involution of the thyroid gland, but volume estimation is also important in stereological studies. None of the aforementioned morphologic methods take into account that the follicle is the functional unit of the thyroid gland. The size of a follicle depends on the size, number and activity of cells, and the amount of colloid [26]. These are changeable and vary with the biological activity of a given follicle and the entirety of the gland. Any change in the size of the thyroid gland could therefore be due to a change in the size of the existing follicles, the number of follicles and amount of follicular epithelia or tissue between the follicles. Examination of histological sections of the human thyroid gland has revealed that the size of the follicles is not homogeneous, as larger follicles are usually found in the periphery and smaller ones are found centrally [27]. However, such information is lacking in dolphins.

Stereological methods have been developed to obtain information about three-dimensional (3-D) structures from 2-D sections of the structures and to achieve information about the whole organ by examining a minor part of it [26], [27]. Direct measurements of the colloid volume and nuclear-to-cytoplasmic (N/C) ratio of the follicular cells might thus provide important information about thyroid gland function, and hormone storage and secretion, which may help understand the changes at morphological and functional levels. In addition, although the knowledge of normal sonographic features and typical follicle structures in human thyroid are well-documented and correlated to each other, such relationship is still lacking in dolphins. The purposes of the present study were to: (a) determine the colloid volume using simple stereological principle and the N/C ratio of bottlenose dolphin's thyroid gland in comparison with the human thyroid gland, and (b) determine how these stereological measurements help understand the dolphin thyroid physiology to aid precise interpretation using ultrasonography and corrective therapy.

## Materials and Methods

### Ethic Statement

This study was licensed under the Animals Control of Experiments Ordinance, Cap 340, issued by the Department of Health of Hong Kong Special Administrative Region. All procedures were reviewed and approved by the Animal Subjects Ethics Sub-committee and the Human Subjects Ethics Sub-committee of the Hong Kong Polytechnic University, and the Scientific Advisory Committee of Hong Kong Ocean Park Corporation. Informed, written consent for research on tissue samples was obtained from each human subject.

### Animals and necropsy procedures

Four Indo-Pacific bottlenose dolphins (2 males and 2 females), which weighed 9.1 kg to 142.1 kg were included in this study. They had been maintained in a semienclosed, outdoor facility, consisting of 5 interconnected tanks with treated natural seawater. Tank capacity was between 100 and 3300 m^3^. Diets consisted of different proportions of capelin, sardine, herring and squid, along with vitamin and mineral supplements, and calorific requirement. Total daily intake was formulated according to individual requirements [28]. The subjects had apparently good clinical histories in the captivity, and had not been receiving medication that would have altered thyroid gland physiology prior to their deceased. Serum concentrations of thyroxine (free [fT4] and total [tT4]), triiodothyronine (free [fT3], total [tT3]) had been determined on each individual subject and the values were all within normal ranges [20]. Histopathology revealed that all dolphin thyroid glands appeared active and normal thyroid cellular architectures were demonstrated.

### Histology processing

At necropsy, thyroid glands were carefully dissected away from the trachea, weighed, and serially sliced at 3-mm intervals while ensuring that all the thyroid tissue were collected and evaluated, and that the necropsy procedure did not damage the tissues. One tissue sample from each thyroid gland was fixed in 10% formalin, processed by conventional techniques, and embedded in paraffin wax at 60°C for histologic processing. Embedded tissue sections (5 µm) were stained with haematoxylin and eosin (H & E). Only one histological section per animal was included in this study. Two human thyroid gland histological sections were also examined for cross species comparison. Each slide was positioned on a light microscope (Olympus CX 40) stage so that the 40× objective lens overlaid a corner of the section before microscopy was started. Fifty follicles were randomly selected by moving the slide under stage micrometer control in increments of 1 mm transversely and vertically, from top to bottom, and captured via a digital camera (Olympus DP 10).

Images were retrieved from the digital camera and transferred to a personal computer. The follicles were measured with a specific quantification software program, ImageJ (National Institute of Mental Health, Bethesda, Maryland, USA). Image magnification was calibrated using a stage micrometer for each objective lens used within this investigation.

### Determination of nuclear-to-cytoplasmic (N/C) ratio

**Figure 1:**
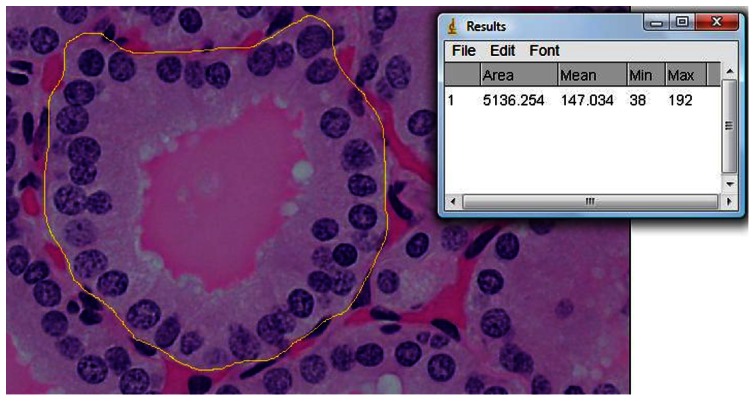
The outer boundary of the follicle was outlined and the area (yellow circle in µm^2^) was measured and recorded.

**Figure 2:**
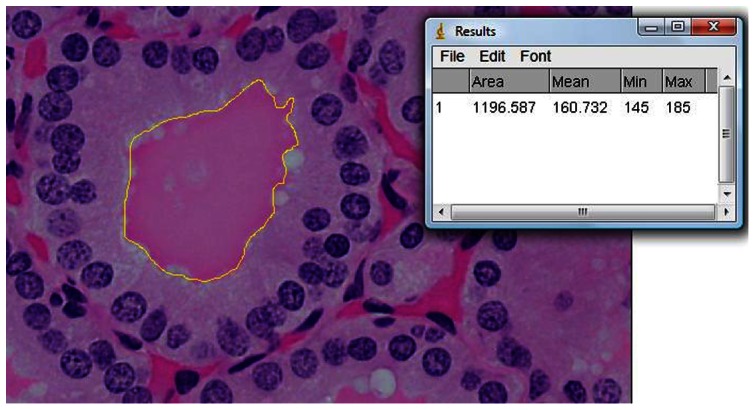
The inner boundary of the follicle was outlined and the area (yellow circle in µm^2^) was measured and recorded.

**Figure 3:**
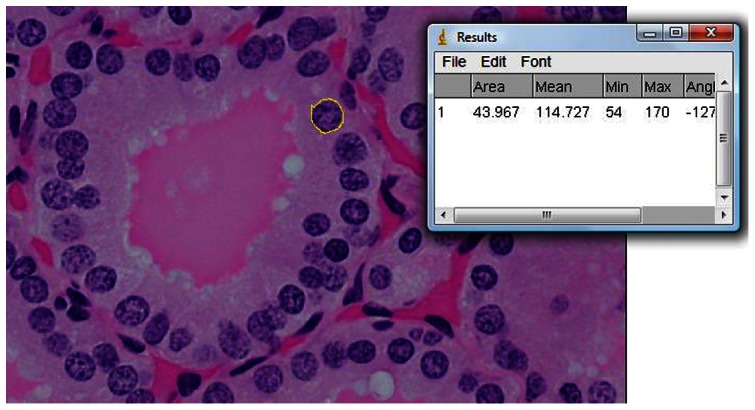
Each nucleus within the cytoplasm was individually outlined and its (yellow circle in µm^2^) was measured and recorded.

Fifty follicles were traced in each subject. In each selected follicle, the outer and inner follicular boundaries were manually traced (Figures 1 & 2). The area of the cytoplasm with nuclei (A_nc_) was determined from the difference of these two boundaries. Subsequently, each individual nucleus within the cytoplasm was also manually traced (Figure 3), with 44 nuclei traced in each follicle on average (ranging from 24 to 68). The areas of these nuclei (A_n_) were summed up, and were subtracted from the original area (A_nc_) to obtain the resultant cytoplasmic area (A_c_).

The nuclear-to-cytoplasmic (N/C) ratio was then determined by the following equation:
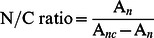



### Determination of colloid volume

**Figure 4:**
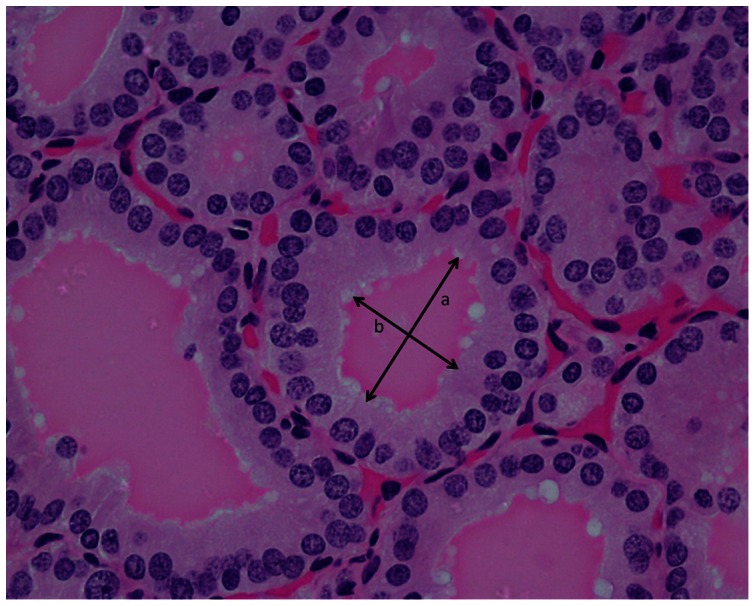
The major axis (longest length) (a) and the minor axis (b) of the colloid was measured and recorded. The major axis (a) is perpendicular to minor axis (b).

In each selected follicle, the major (*a*) and minor (*b*) axes were measured manually from the colloid area (Figure 4). The major axis (*a*) is perpendicular to minor axis (*b*).

Direct measurements of *a* and *b* were converted to the diameter of a circle of equivalent area (d) by using the formula:
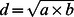
where *a* and *b* are the major and minor axes of the colloid area respectively. To compensate for the effects of sectioning a sphere, the measured diameter (d), which is an underestimation, was corrected by the method of Abercrombie [29] to provide an estimate of the mean diameter (Ð),
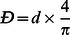



The corrected mean diameter (Ð) was used to determine the volume of the colloid area (Vcol) on the assumption that the colloid profiles had been converted to circles of equivalent areas by the formula:
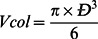



### Statistical analysis

The mean colloid volume and N/C ratio from both dolphin and human subjects were cross-compared with the Mann-Whitney U-test (GraphPad InStat, GraphPad Software, Inc., San Diego, CA, USA). Kruskal-Wallis test (non-parametric ANOVA) was used to calculate the level of significance of the variation in colloid volume and N/C ratio among all dolphin subjects and Dunn post-test was used as the posthoc test, for each available dolphin subject. A p value<0.05 is considered as statistically significant.

## Results

The four animals included in the study are listed in Table 1, with details of their age, sex, body length, thyroid weight and reported the cause of death. The thyroid gland was located at the lateral-ventral aspect of the thyroid cartilage. No gross lesions were observed among the four dolphins.

**Table 1:** Demographic parameters and cause of death of the Indo-Pacific bottlenose dolphin subjects.

Subject	Sex	Sexual Maturity	Weight at death	Estimated Age at death	Thyroid weight at necropsy	Cause of death
D1	M	Sexually immature	9.1 kg	4 days	N/A	malnutrition, hypoglycemia, drowning
D2	F	Sexually mature	112 kg	7–10 years	58 g	Melioidosis
D3	M	Sexually mature	142.1 kg	24 years	20 g	acute hepatic necrosis with an unidentified cause
D4	F	Sexually mature	137 kg	37 years	20 g	chronic obstrcutive pulmonary diseases, aged

**Figure 5:**
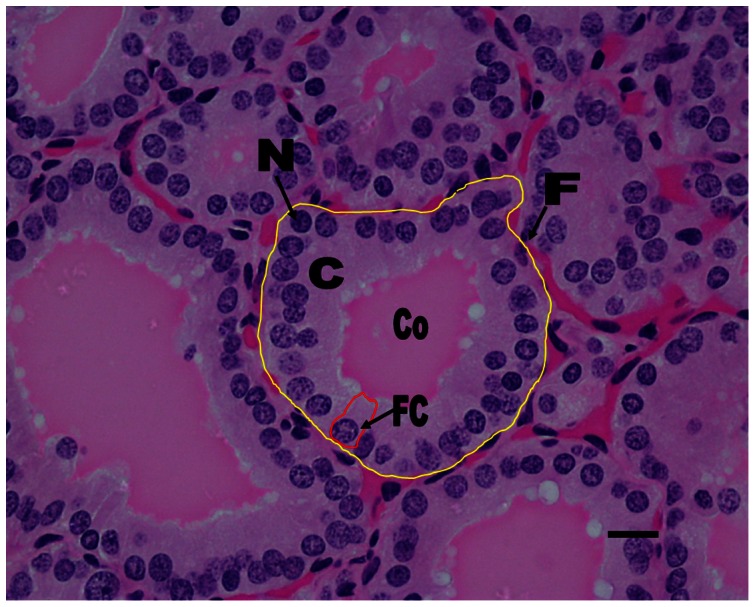
This figure showed a normal dolphin's thyroid gland section with Haematoxylin and Eosin Stain. The thyroid follicle (F) (outlined in yellow solid line) were lined by a single layered columnar follicular cell (FC) (outlined in red solid line) with round nuclei (N). The lumen contained homogeneous colloid (Co) material. Bar, 20 µm.

**Figure 6:**
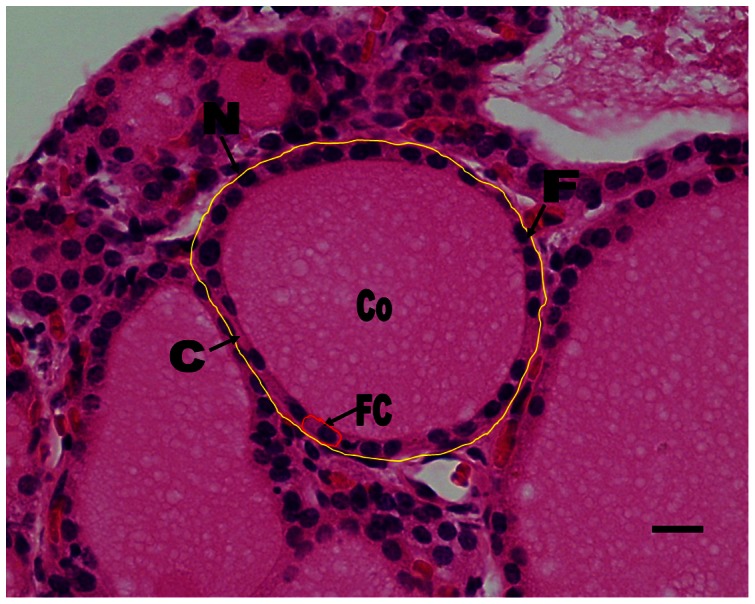
This figure showed a normal human thyroid gland section with Haematoxylin and Eosin Stain. The thyroid follicle (F) (outlined in yellow solid line) were lined by a single layered cuboidal follicular cell (FC) (outlined in red solid line) with round nuclei (N). The lumen contained homogeneous colloid (Co) material. Bar, 100 µm.

Histologically, thyroid follicular epithelia were more cuboidal in human glands than in dolphins, which were generally columnar. In both dolphin and human thyroid glands, the size of the follicles tended to be variable (Figures 5 & 6). The distribution of large and small follicles within the thyroid gland was also random in both the dolphin and human thyroid glands. However, the dolphin follicular epithelia may be more columnar with age. Result showed that there was no significant difference (p>0.05) in colloid volume among all the dolphins; however, the size of follicles appeared to decrease as a function of increasing age in the dolphin thyroid gland. Kruskal-Wallis test revealed that there were significant differences (p<0.0001) in N/C ratios among all the dolphins; however, Dunn post-tests illustrated that there was no significant difference between dolphin 1 (D1) and dolphin 2 (D2) as well as D2 and dolphin 3 (D3).

The mean colloid volume of the dolphin thyroid gland and human thyroid gland was 1.22×10^5^ µm^3^ and 7.02×10^5^ µm^3^ respectively. The dolphin and human subjects had a significant difference in the mean colloid volume (p<0.0001). The mean N/C ratio of the dolphin thyroid gland and human thyroid gland was 0.50 and 0.64 respectively. The dolphin and human subjects had a significant difference in the mean N/C ratio (p<0.0001).

**Figure 7:**
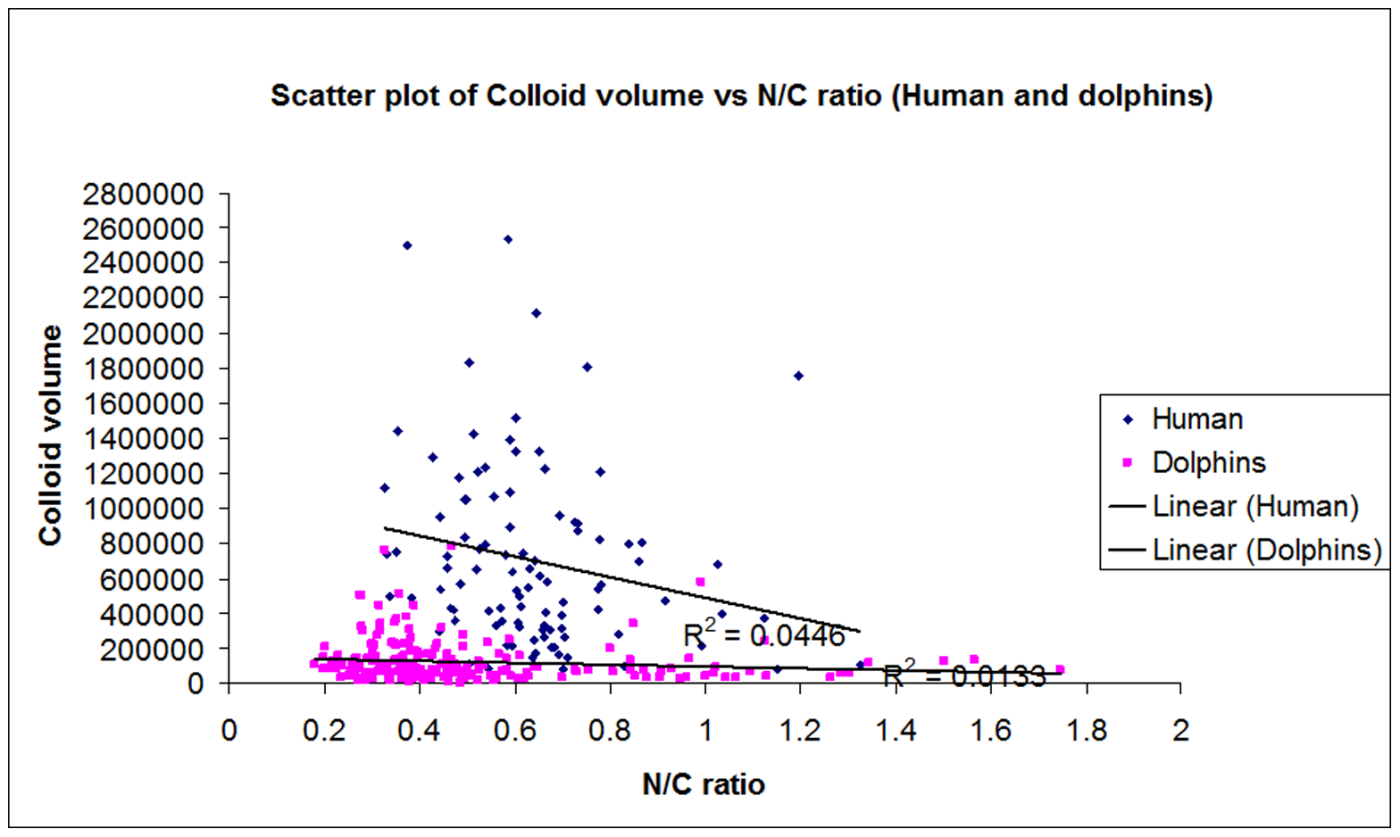
Scatter plot of colloid volume against N/C ratio in dolphin and human subject.

Figure 7 showed the scatter plot of colloid volume against N/C ratio in dolphin and human subject. The colloid volume of human thyroid gland was greater than the dolphin thyroid gland as previously observed. The N/C ratio of human thyroid gland appeared more constant (ranged from 0.33 to 1.33) while that of the dolphin gland appeared more variable (ranged from 0.18 to 1.75). Trend lines of both human and dolphin data were drawn and no correlation between the colloid volume and N/C ratio was found in both human and dolphin thyroid gland.

## Discussion

This study showed that there was no significant difference in thyroid gland histology between dolphin and humans, which is consistent with the previous investigations [12],[30]. However, using simple stereological principles and calculations, two important variables in the description of the thyroid morphology were calculated for dolphin and human subjects: the colloid volume and the N/C ratio, which may provide considerable insight to the thyroid function.

The total volume of the colloid in each follicle is a measure of the storage capacity and the follicular function of the gland as a whole [16], whereas the N/C ratio is a measure of the metabolic activity of the follicular cells. In the present study, the mean colloid volume of the dolphin's thyroid gland was approximately 1/6 of that of the human thyroid gland, which was significantly reduced. The N/C ratio of the dolphin thyroid follicular cells was also significantly smaller than human thyroid gland. Previous studies revealed that bottlenose dolphins have nearly twice as much thyroid gland proportionately in weight to humans (400 mg/kg vs 250 mg/kg) [5]. Since thyroid hormones regulate a wide variety of metabolic processes, particularly those associated with energy metabolism, the finding of relatively high circulatory levels of THs in some cetaceans compared with humans and other terrestrial mammals have reinforced the long standing notion of elevated metabolic rates in cetaceans. This discrepancy in the colloid volume of dolphin and human subjects accounts for the differences in metabolic rates. Higher metabolic rates in the dolphin was associated with and induced by an increase in TSH production, resulting in a higher level of secretion of thyroid hormone. The histological appearance of the thyroid gland is dramatically influenced by TSH [31]. When TSH secretion increases, the first response is formation of numerous cytoplasmic pseudopods, which result in increased endocytosis of thyroglobulin-rich colloid from the follicular lumen [32]. If increase TSH secretion is sustained, the colloid volume will become smaller because of increased endocytosis of the colloid [31],[33] with numerous periodic acid-Schiff (PAS) positive colloid droplets present along the luminal side of hypertrophic follicular cells. In the dolphin's thyroid gland, the follicular cells were more columnar than those in human thyroid gland. Assuming that there was no significant difference in the nuclear area of both species, then the area occupied by the cytoplasm in dolphin thyroid follicular cells would be larger than that of the humans to yield a smaller N/C ratio on average. Changes in the cytoplasmic volume can occur due to a number of reasons, such as volume overload in cardiac hypertrophy, osmotic stress or other signals. These in turn affect a variety of cellular responses, including metabolism and gene regulation [34],[35]. Thus the smaller N/C ratio may imply the existence of a higher metabolic rate in dolphins when compared to human subjects.

Another factor which may impact colloid volume discrepancy is stress in a captive environment. The dolphin subjects included in the present study had been living in a captive environment from 4 days up to 24 years. Stress from captivity as well as being trained for displaying acrobatics, although rewarded by food, may cause increased TSH production from the anterior pituitary gland which is associated with increased metabolic rates. [36]. The dolphin's thyroid gland in the present study all showed signs of increased activity consistent with mild TSH stimulation, compared to the healthy adult male human subject observed. Other studies which had also reported that altered THs status have been associated with capture and handling in belugas [37],[38] suggested initial stress-mediated changes in hormone secretion and metabolism may cause the cortisol to inhibit TSH secretion and also the monoiodinase responsible for producing much of the T3 in circulation; these pathways appeared to be different in the bottlenose dolphins.

To our knowledge, this is the first time that stereological approach has been applied to the colloid volume measurement of the cetacean thyroid gland. Direct comparison between the findings of the present study and previous dolphin histological studies may be difficult; however, colloid volume may indirectly reflect the average follicle diameter, which has been consistently documented in previous histology studies in dolphin. A study reported that the thyroid gland of long-finned pilot whales exhibited noticeable variation in size and appearance with advancing age, although active development of new follicles was reported in all stages of development in Pacific white-sided dolphins [36]. Generally speaking, the thyroid follicles are more regular in young animals. In cetaceans, colloid is slightly chromophile, its appearance is more pale and fluid, and the epithelia are tall. In older animals, the colloid is less hydrated and a large number of lamellar basophile masses are present in the thyroid gland [39]. This observation was also noted in the present study, with larger volumes of colloid surrounded by the more columnar follicular cells in younger animals and a smaller amount of colloid surrounded by the more cuboidal follicular cells in older animals. However, no statistically significant difference was found among the four dolphin subjects, possibly due to the small sample size in the present study. Further study should be done with more subjects included in each age group for comparison purposes.

In the present study, a significant difference in the N/C ratio was found among dolphin subjects in different age classes, which may reflect the difference in metabolic activity of the follicular cells with stages of development in dolphins (Table 1). An experimental animal study involving fasted and refed male Wistar rats found age-related changes in the structure and function of thyroid follicular cells [40]. Older rats had smaller follicular epithelium and smaller colloid resorption than young rats. In humans, thyroid gland tissue was found to be more morphologically active in children than in adults [41]. Younger children had smaller follicles and higher expression of proteins involved in iodine transport, indicating higher thyroid activity and higher thyroid cell proliferation in connection with pubertal growth. A decrease in N/C ratio in dolphin's thyroid gland with advancing age was observed in the present study which may indicate decreased thyroid function with advancing age, similar to humans and rats. Further study should be undertaken with more dolphin subjects included in each age group to investigate possible discrepancies in the metabolic activity of thyroid gland.

Studies also suggested that the average follicle diameter of the thyroid gland in wild dolphin populations is larger than that of captive dolphin populations (200 µm vs 50 µm) [38]. Necropsy reports of captive dolphins found that thyroid follicles were uniformly small, with an average diameter of 30–50 µm, with columnar epithelia and condensed colloid. Many follicles contained only colloid remnants, suggestive of severe thyroid gland depletion. A similar case was also found in the 3 bottlenose dolphins in this study, with varying degrees of colloid depletion. Sick dolphins in captivity, despite all efforts to keep them eating, become anorexic. It is suggested that their diseases may have incapacitated them to obtain food or keep up with companions because of reduced swimming speed. Parasitic infections or lung diseases may also interfere with the production of sonic and ultrasonic pulses or clicks and thus affects echolocation for navigation and food finding purposes [42]. Although it is difficult to imagine how a marine animal living in an environment of ocean water containing a relatively large amount of iodine could develop a dietary deficiency of this element. The low plasma values for total iodine and inorganic iodine seem to indicate impairment in absorption, a dietary deficiency or a more rapid utilization and thus faster turnover rate. Further studies are needed to investigate the effects of progressive thyroid gland depletion in captive and stranded dolphin populations.

The discrepancy of the colloid volume in dolphin and human subjects may also be substantiated by differences in echogenicity of the thyroid glands with ultrasonography. Echogenicity of the thyroid gland varied among individuals as well as different lobes or portions of a single dolphin's thyroid gland. Human thyroid gland appeared hyperechoic when compared to the sternocleidomastoid muscle. Changes in echogenicity could represent different thyroid gland abnormalities such as subclinical hypothyroidism and Hashimoto's thyroiditis [43],[44]. The normal echogenicity of the thyroid gland parenchyma is determined by the typical follicle structures [45]. The interface between thyroid gland cells and the colloid exhibit high acoustic impedance, causing high frequency acoustic waves to be reflected back to the transducer. The present study proved that the dolphin thyroid gland cells were found to be smaller than the normal human thyroid gland cells, resulting in a lower echogenicity [23]. A dolphin thyroid gland with hypoechoic and isoechoic appearances is thus considered to be normal. In addition, varied echogenicity at different lobes or portions of a single thyroid gland may be explained by uneven distribution of various sizes of follicles within the single dolphin's thyroid gland. Shimokawa et al. [46] reported that irregular or oval follicular lumens were seen in the parenchyma of the thyroid gland, with the size of the follicular lumen appearing larger in the central regions than in the peripheral regions in Risso's dolphin.

It is important to note that although the present study reported the colloid volume and N/C ratio of the dolphin and human subjects, it is well known that soft tissues shrink during histological processing. Thus the colloid volume estimated in this study may not reflect the *in vivo* quantities. The N/C ratio was expressed as a ratio which should not affected by shrinkage. Also, in the present study, small sample size and unknown age and health status of the human subjects may not necessarily represent the overall thyroid gland function in dolphins and humans. A larger sample size of dolphin and human subjects with known age and sex match, with more tissue samples collected and evaluated from each individual is suggested to facilitate a more direct and comprehensive cross-comparison between the two species.

## Conclusions

This stereological study compared the colloid volume and the N/C ratio between dolphin and human thyroid gland. These findings provide valuable insight into dolphin thyroid physiology, and aid precise diagnosis using ultrasonography and corrective therapy in live subjects. Further investigation on the morphological changes, other than the variation in size in dolphin's thyroid gland, should be undertaken during different seasons, physiological and pathological conditions for comparison purposes.

## References

[1] Norris DO (2007) Vertebrate endocrinology. 4th ed. Boston: Elsevier Academic Pres. 560 p.

[2] Hegedus L (2001) Thyroid ultrasound. Endocrinol Metab Clin North Am 30: 339–360. 11444166 10.1016/s0889-8529(05)70190-0

[3] Myers MJ, Rea LD, Atkinson S (2006) The effect of age, season and geographic region of thyroid hormones in Steller sea lions (*Eumetopias jubatus*). Comp Biochem Phys A 145: 90–98. 10.1016/j.cbpa.2006.05.004 16815718

[4] Foktin J, Matragrano J, Ransom J, Davis K (2003) Review of Medical Physiology. 23th edition. McGraw-Hill Companies. p.

[5] St. Aubin DJ (2001) Endocrinology. In: Dierauf LA, Gulland MD, editors. CRC handbook of marine mammal medicine: Health, disease and rehabilitation. Boca Raton: CRC Press. pp. 165–192.

[6] Eales JG (1988) The influence of nutritional state on thyroid function in various vertebrates. Am Zool 28: 351–362.

[7] Porterfield S (1994) Vulnreability of the developing brain to thyroid abnormalities: Environmental insults to the thyroid system. Environ. Health Perspect 102: 125–130. 10.1289/ehp.94102125 PMC1567088 7925183

[8] Porterfield S, Stein S (1994) Thyroid hormones and neurological development: Update. Endocr Rev 3: 357–363. 10.1210/edrv-14-1-94 8491157

[9] Harrison RJ, Rowlands IW, Whitting HW, Young BA (1962) Growth and structure of thyroid gland in the common seal. J Anat 96: 3–15. 13904910 PMC1244168

[10] St. Aubin DJ, Geraci JR (1989) Seasonal variation in thyroid morphology and secretion in the white whale. Can J Zool 67: 263–267.

[11] St. Aubin DJ, Ridgway SH, Wells RS, Rhinehart H (1996) Dolphin thyroid and adrenal hormones: circulating levels in wild and semi-domesticated *Tursiops truncates*, and influence of sex, age, and season. Mar Mammal Sci 12: 1–13.

[12] Ridgway SH, Patton GS (1971) Dolphin thyroid: Some anatomical and physiological findings. Z Vgl Physiol 71: 129–141.

[13] Harrison RJ, Young BA (1970) The thyroid gland of common Pacific dolphin, Delphinus delphis bairdi. J Anat 106: 243–254. 5442223 PMC1233699

[14] Irving L, Scholander PF, Grinnell SW (1941) The respiration of the porpoise, *Tursiops truncatus* . J Cell Comp Physiol 17: 145–168.

[15] Van Dyke D, Ridgway SH (2001) Nutrition. In: Dierauf LA, Gulland MD, editors. CRC handbook of marine mammal medicine: Health, disease and rehabitation. Boca Raton: CRC Press. pp. 595–598.

[16] Hartoft-Nielsen ML, Rasmussen AK, Feldt-Rasmussen U, Buschard K, Bock T (2005) Estimation of number of follicles, volume of colloid and inner follicular surface area in the thyroid gland of rats. J Anat 207: 117–124. 16050898 10.1111/j.1469-7580.2005.00442.x PMC1571517

[17] Conde E, Martin-Lacave I, Gonzalez-Campora R, Galera-Davidson H (1991) Histometry of normal thyroid glands in neonatal and adult rats. Am J Anat 19: 384–390. 10.1002/aja.1001910405 1951136

[18] Amoroso EC, Bourne GH, Harrrison RJ, Matthews LH, Rowlands IW (1965) Reproductive and endocrine organs of foetal, newborn and adult seals. J Zool 147: 430–486.

[19] Little GJ (1991) Thyroid morphology and function and its role in thermoregulation in the newborn southern elephant seal at Macquarie Island. J Anat 64: 97–106. PMC1260313 1917675

[20] Hall AJ, Green NJL, Jones KC, Pomeroy PP, Harwood J (1998) Thyroid hormones as biomarkers in grey seals. Mar Pollut Bull 36: 424–428.

[21] Brook F (1997) The use of diagnostic ultrasound in assessment of the reproductive status of bottlenose dolphin, *Tursiops aduncas*, in captivity and applications in management of a controlled breeding program, Ph.D. thesis. Hong Kong Polytechnic University, Kowloon, Hong Kong.

[22] Kot BCW, Ying MTC, Brook FM, Kinoshita RE (2012) Evaluation of two-dimensional and three-dimensional ultrasound in the assessment of thyroid volume of the Indo-Pacific bottlenose dolphin (*Tursiops aduncus*). J Zoo Wildlife Med 43: 33–49. 10.1638/2010-0190.1 22448508

[23] Kot BCW, Ying MTC, Brook FM, Kinoshita RE, Cheng SCH (2012) Sonographic assessment of the thyroid gland and adjacent anatomic structures in Indo-Pacific bottlenose dolphin (*Tursiops aduncus*). Am J Vet Res In press. 10.2460/ajvr.73.11.1696 23106453

[24] Kot BCW, Ying MTC, Brook FM, Kinoshita RE (2012) Sonographic evaluation of thyroid morphology during the normal estrous cycle in the Indo-Pacific bottlenose dolphin (*Tursiops aduncus*). J Zoo Wildlife Med 43: 256–264. 10.1638/2010-0196.1 22779228

[25] Kot BCW, Ying MTC, Brook FM, Kinoshita RE, Dave K, et al (2011) Sonographic evaluation of thyroid morphology during different reproductive events in female Indo-Pacific bottlenose dolphins, *Tursiops aduncus* . Mar Mamm Sci In press.

[26] Charles CC, Kovacs K, Horvath E, Stefaneanu L (1996) Anatomy. In: Braverman LE, Utiger RD, editors. Werner and Ingbar's the Thyroid. A fundamental and clinical text. Philadelphia: Lippincott-Raven. pp. 19–46.

[27] Brown RA, Al-moussa M, Beck JS (1986) Histometry of normal thyroid in man. J Clin Pathol 39: 475–482. 3722401 10.1136/jcp.39.5.475 PMC499907

[28] Gallego P (2000) Food management in bottlenose dolphins (*Tursiops truncates*), Ph.D. thesis. University of Liège, Wallonia, Belgium.

[29] Abercrombie M, Johnson ML (1946) Quantitative histology of Wallerian degeneration: I. Nuclear population in rabbit sciatic nerve. J Anat 80: 37–50. PMC1272751 20996672

[30] Cowan DF, Tajima Y (2006) The thyroid gland in bottlenose dolphins (Tursiops truncatus) from the Texas Coast of the Gulf of Mexico: Normal structure and pathological changes. J Comp Path 135: 217–225. 17034811 10.1016/j.jcpa.2006.07.003

[31] Collins WT, Capen CC (1980) Ultrastructural and functional alterations of the rat thyroid gland produced by polychlorinated biphenyls compared with iodide excess and deficiency, and thyrotropin and thyroxine administration. Virchows Arch 33: 213. 10.1007/BF02899183 6110270

[32] Nilsson M, Ericson LE (1986) Graded response in the individual thyroid follicle cell to increasing doses of TSH. Mol Cell Endocrinol 44: 165. 3949068 10.1016/0303-7207(86)90059-6

[33] Ramsdem JD (2000) Angiogenesis in the thyroid gland. J Endocrinol 166: 475. 11029748 10.1677/joe.0.1660475

[34] Oney I, Kurnaz IA, Kurnaz ML (2005) Cytoplasmic-to-nuclear volume ratio affects AP-1 complex formation as an indicator of cell cycle responsiveness. FEBS Letters 579: 433–440. 15642355 10.1016/j.febslet.2004.11.104

[35] Ronsch H, Kramer R, Morbach S (2003) Impact of osmotic stress on volume regulation, cytoplasmic solute composition and lysine production in Corynebacterium glutamicum MH20-22B. J Biotechnol 104: 87–97. 12948632 10.1016/s0168-1656(03)00166-4

[36] Harrison RJ (1969) Endocrine organs: Hypophysis, thyroid and adrenal. In: Andersen HT, editor. The biology of marine mammals New York: Academic Press. pp. 349–390

[37] St. Aubin DJ, Geraci JR (1988) Capture and handling stress suppresses circulating levels of thyroxine and triiodothyronine in beluga whales, *Delphinapterus leucas*,. Physiol Zool 61: 170–175.

[38] St. Aubin DJ, Geraci JR (1992) Thyroid hormone balance in beluga whales, *Delphinapterus leucas*: Dynamics after capture and influence of thyrotropin. Can J Vet Res 56: 1–5. 1586888 PMC1263494

[39] Arvy L (1970) Endocrine glands and hormonal secretion in cetaceans, In: Pilleri G, editor. Investigations on Cetacea. Switzerland: Berne. pp 230–251.

[40] Kmieć Z, Kotlarz G, Smiechowska B, Myśliwski A (1998) The effect of fasting and refeeding on thyroid follicule structure and thyroid hormone levels in young and old rats. Arch Gerontol Geriatr 26: 161–175. 18653134 10.1016/s0167-4943(97)00040-x

[41] Faggiano A, Coulot J, Bellon N, Talbot M, Caillou B, et al (2004) Age-dependent variation of follicular size and expression of iodine transporters in human thyroid tissue. J Nucl Med 45: 232–237. 14960641

[42] Geraci JH, St Aubin DJ (1987) Effects of parasites on marine mammals. Int J Parasitol 17: 407–414. 3294652 10.1016/0020-7519(87)90116-0

[43] Schiemann U, Avenhaus W, Konturek JW, Gellner R, Hengst K, et al (2003) Relationship of clinical features and laboratory parameters to thyroid echogenicity measured by standardized grey scale ultrasonography in patients with Hashimoto's thyroiditis. Med Sci Monit 9: 49–53. 12709678

[44] Vejbjerg P, Knudsen N, Perrild H, Laurberg P, Pedersen IB, et al (2006) The association between hypoechogenicity or irregular echo pattern at thyroid ultrasonography and thyroid function in the general population. Eur J Endocrinol 155: 547–552. 16990653 10.1530/eje.1.02255

[45] Müller HW, Schröder S, Schneider C, Seifert G (1985) Sonographic tissue characterisation in thyroid gland diagnosis. A correlation between sonography and histology. Klin Wochenschr 63: 706–710. 3900555 10.1007/BF01733114

[46] Shimokawa T, Nakanishi I, Hondo E, Iwasaki T, Kiso Y, et al (2002) A morphological study of the thyroid gland in Risso's Dolphin, Grampus griseus. J Vet Med Sci 64: 509–512. 12130836 10.1292/jvms.64.509

